# Supplementary material for: Regional anesthesia educational material utilization varies by World Bank income category: A mobile health application data study
Source: PLoS One. 2021 Feb 1;16(2):e0244860. doi: 10.1371/journal.pone.0244860 (PMC7850494; doi:10.1371/journal.pone.0244860)

S1 Fig. Screenshot of the app.

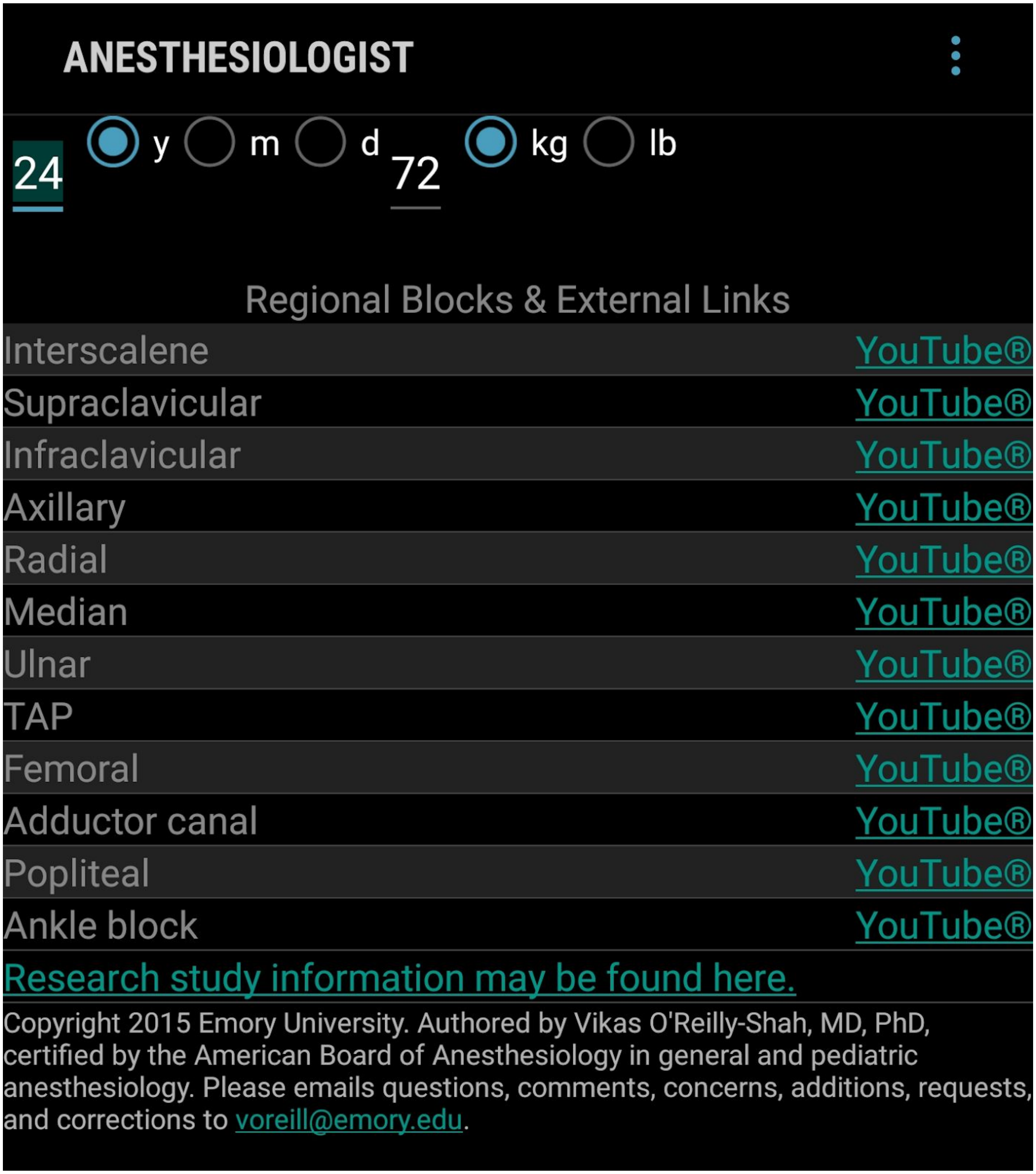

Supplement: S1 Fig — (PDF) [file pone.0244860.s001.pdf]
