# Supplementary material for: Regional anesthesia educational material utilization varies by World Bank income category: A mobile health application data study
Source: PLoS One. 2021 Feb 1;16(2):e0244860. doi: 10.1371/journal.pone.0244860 (PMC7850494; doi:10.1371/journal.pone.0244860)
Supplement: S1 File — (PDF) [file pone.0244860.s002.pdf]

S1 File. Description of the app, including links to the app

The app "Anesthesiologist" was designed to help quickly calculate adult and pediatric anesthesia-related information like common drug dosing or airway related information such as endotracheal tube size. The app was written solely to be used as a helpful adjunct for professionally trained physicians and practitioners otherwise experienced in airway management and drug administration and dosing. There is no peer review of the app in the classical sense; the app functions much as a calculator and not as guidelines. The anesthesiologist app does provide crowdsourced educational material in the form of links to nerve blocks available publicly on YouTube (videos peer-reviewed by the app's author) and it crowdsources information by asking professionals to voluntarily fill out surveys. The app can be found here:
https://play.google.com/store/apps/details?id=com.shahlab.anesthesiologist&hl=en_US
