# Supplementary material for: Regional anesthesia educational material utilization varies by World Bank income category: A mobile health application data study
Source: PLoS One. 2021 Feb 1;16(2):e0244860. doi: 10.1371/journal.pone.0244860 (PMC7850494; doi:10.1371/journal.pone.0244860)
Supplement: S2 File — (PDF) [file pone.0244860.s003.pdf]

S2 File. Survalytics Detailed Description

The Survalytics platform is designed to send survey questions to the app and to retrieve survey responses and other analytic metadata from the app. These surveying capabilities are not one-time or static. New survey questions can be delivered via the Internet to the installed base of mobile devices, with the questions being presented to the app users the next time the app is opened. Survey data and app usage information are transmitted to and from the app utilizing services provided "in the cloud" by Amazon Web Services (Amazon Seattle, WA).

A detailed schema for the survey and analytic data collection was developed. The Survalytics platform allows for the surveys to have a branched structure. Such a branched survey was used to collect basic demographic information from the user after initial installation and agreement by the user to participate in the study. The survey questions are summarized in Table S1. Users could opt in or opt out of the study at any time.

Location of the device was determined using three different approaches, as described below. For all of the approaches, only the country and "administrative region" were determined and stored, even when more precise determination of location was possible. Here "administrative region" refers to the largest geographical subdivision within the country such as the state in the U.S. or province in India. The precision of the location determination was limited to granularity no more defined than administrative region to provide Health Insurance Portability and Accountability Act (HIPAA) compliant de-identification of data. Healthcare providers were entering into the app a patient age and weight. If the location information stored were more precise, patient age and weight information entered into the app might be combined with the specific location and date in a manner that could potentially comprise protected health information (PHI) as defined by HIPAA.

The first of the three approaches to determining the country and administrative region data was based on GPS coordinates which were reverse geocoded using Google's Geocoding API [1]. “Reverse geocoding” refers to the process of converting longitude and latitude coordinates, such as those provided by GPS, into human-interpretable geographic descriptions such as country, state/province, or address. The second approach was based on using the mobile device’s Internet Protocol (IP) address. The IP address was reverse geocoded using a web-based service provided by ip-api.com [2]. The last approach was based on the country code stored in the memory chip used to uniquely identify the device (the Subscriber Identity Module or SIM card). Only country information is available via this last approach.

During analysis, the country and administrative region from GPS reverse geocoding was preferentially used. However, GPS coordinates were not always available for a variety of reasons including GPS reception problems, GPS sensor failure, or the device user not consenting to sharing GPS location information. If GPS data were not available, the country and administrative region from IP address was used. Sometimes, this information was not available due to lack of Internet connectivity at the time of data collection. If not, the country from the SIM card (felt to be the least accurate) was used.

The Survalytics platform stores each "event" (e.g. consent, a survey response, an in-app click, or closure of the app) in a local database on the device. When Internet connectivity is detected, one data packet is transmitted from the app at a time, with each packet representing a single "event". Each packet contains relevant details of the event (e.g. what was clicked), as well as a generic set of information including an anonymous globally unique identifier (generated when the app is first opened on the device), time information (specifically, timestamp, time zone, and local time), location information (from the three sources outlined above), and device

language. Transmitted packets are stored as records in an Amazon Web Services DynamoDB database. See the publication describing Survelytics [3] for even further additional technical details.

The anonymous user identifier allows for all of the data from one device to be tied together. Together with the time stamps, this allows the sequence of app usage events and survey responses for each mobile device to be reconstructed from the database.
