# Supplementary material for: Regional anesthesia educational material utilization varies by World Bank income category: A mobile health application data study
Source: PLoS One. 2021 Feb 1;16(2):e0244860. doi: 10.1371/journal.pone.0244860 (PMC7850494; doi:10.1371/journal.pone.0244860)
Supplement: S3 File — (PDF) [file pone.0244860.s004.pdf]

S3 File. Mobile Healthcare App Study Database Schema

I. Survey/demographics central database tables

The overall architecture is designed to simplify the codebase by using JSON primarily as a transport vehicle and limiting the number of database fields to those that need to be known by the database in question. For example, the AWS source database for downloading questions only needs to know question guide (for a hash key) and the json_str containing the meat of the question. Telling it ordinal position simplifies other areas of the Android code and so that was included. Otherwise, the content remains unparsed until downloaded by the Android app.

On device, the database is again limited to guid, ordinal position, and jsonstr. The additional fields are flags for internal tracking use. Parsed JSON supplies fields for the generation of the question on-device and for the uploaded response.

http://www.jsoneditoronline.org/
https://www.guidgenerator.com/online-guid-generator.aspx

On AWS: Question Table:

```
    questionguid_str        : STRING, PRIMARY HASH KEY
    ordinalposition_int     : INT, RANGE KEY
    json_str                : STRING
```

json_str JSON Schema: Question

```
{
    surveyname_str          : STRING
    surveyguid_str: STRING
    ordinalposition_int     : INT
    questionguid_str        : STRING
    questionprompt_str      : STRING
    questiontype_str        : STRING
    responses_arr  : ARRAY
        [
            {
            responseid_int          :INTEGER
            response_str            :STRING
            },
            {
            responseid_int          :INTEGER
            response_str            :STRING
            },
            ....
        ]
OPTIONALLY
    conditional_upon_questionguid_str   : STRING // questionguid to check*
    conditional_upon_responseid_int     : INTEGER // response id to check*
```

```
            //*-above two work together and both required to be specified

    conditional_upon_datemsid_int         : INTEGER
            // date (in UTC Unix epoch ms) after which to administer this question

    conditionalbycountry_str              : STRING // use ISO 3166 alpha-2 codes

    delaybydays_int                       : INTEGER
            //wait this many days after the question is first downloaded to ask this question

    ongoingquestion_arr                   : ARRAY //array of day of week+time as follows
            [
                    {
                    notificationtime_str    : STRING
                     },
                    {
                    notificationtime_str    : STRING
                     },
                    ...
                    //notificationtime formatted as follows:  EEEHHmm
                    // EEE = three letter day of week (Mon, Tue, Wed, Thu, Fri, Sat, Sun,
Dly)
                    //                                      Dly = daily
                    // HH = military time hours 00-23
                    // mm = minutes 00-59
                    //  Examples:      Tue0900, Thu1400, Dly1200
            ]

    deletequestion_str                    : STRING //questionguid of ongoing question to
                                                  // delete from local SQLite db

}
```

Local DB on Android

Table questions
questionguid_str
json_str
ordinalposition_int   //Primary key
final_responseid_int
final_response_str
answered_bool
uploaded_bool //unused

Table responses
_id

json
uploaded

II. Responses: Generic schema

The generic schema serves as the basic information passed with all types of uploaded data. The additional overhead is minimal and the presence of this information in each uploaded packet simplifies future analysis against unnecessary complexity in terms of cross references and joins.

```
{
	userguid_str			: STRING	PRIMARY RANGE INDEX
	localtime_ms_int		: INTEGER	PRIMARY HASH INDEX
	localtime_hrsmilitary_int	: INTEGER
	localtime_dayofweek_str		: STRING
	localtimezone_str		: STRING
	country_tm_str			: STRING
	lo_lang_str			: STRING	//locale lang
	app_lang_str			: STRING
	region_ipapi_str		: STRING	//www.ip-api.com/json
	regionname_ipapi_str		: STRING
	country_ipapi_str		: STRING
	region_gc_str			: STRING	//geocoding
	country_gc_str			: STRING
	entrytype_str			: STRING	// included in all section III items
	...
}
```

III. Responses: Specific added fields to generic document schema

Survey/demographics data

```
…
entrytype_str            : “survey”,
surveyguid_str           : STRING
questionguid_str         : STRING
questionprompt_str       : STRING
response_str             : STRING
responseid_str : STRING //questionguid & "-" Integer.toString(respid)
responses_arr  : ARRAY [if type is multiple response eg checkbox)
        [
                {
                responseid_str          :STRING
                                //questionguid & "-" Integer.toString(respid)
                response_str            :STRING
                },
                {
                responseid_str          :STRING
                                //questionguid & "-" Integer.toString(respid)
                response_str            :STRING
                },
                ....
        ]
```

Consent/Consent Change

```
...
entrytype_str            : “consentcode_int/consentchange_int”
“consentcode_int”        : INTEGER
“consentchange_int”      : INTEGER

                                1 - do not consent
                                2 - consent
                                3 - exit study
                                4 - re-enter study
```

On Start

```
...
entrytype_str                   : “onstart”
“age_yrs_fra”                   : FRACTION
“weight_kg_fra”                 : FRACTION
```

Age/weight entered by app user (age over 89 to be reported as 89+)

```
...
entrytype_str               : “ageweight”,
“age_yrs_fra”               : FRACTION
“weight_kg_fra”             : FRACTION
```

Total time using the app

```
...
entrytype_str               : “totaltimeofuse”,
“timeinapp_ms_int”          : INTEGER,
"ageweightmodified_int      : INTEGER //0=no 1=yes
```

Drugs favorited and changes to favorites

```
…
entrytype_str               : “favoriteslist”,
“favoriteslist_arr”         : ARRAY
    [
        {   “drugid_int” : drug.get_id(),           INTEGER
            “name_str”: drug.getDrugName(),         STRING
            “position_int” : favepos                INTEGER
        } ,
        {   “drugid_int” : drug.get_id(),           INTEGER
            “name_str”: drug.getDrugName(),         STRING
            “position_int” : favepos                INTEGER
        } ,
        ....
    ]
```

In-app clicks (drugs, Epocrates, airway setup guide, critical events checklist, externally linked nerve blocks)

```
...
entrytype_str   :       See the click types below

Entrytype_str click types:
    “drugclick”,
    “epocrates”,
    “linkline_str”,
    “airwaysetupguide”

Extra JSON for drug/epocrates
    “drugid_int”                : drug.get_id()
```

“name_str” : drug.getDrugName()

Extra JSON for linkline:

“linkline_str” : STRING == name //nerveblock and spachecklist
“linklineurl_str” : STRING == link //nerveblock and spachecklist
