# Supplementary material for: Regional anesthesia educational material utilization varies by World Bank income category: A mobile health application data study
Source: PLoS One. 2021 Feb 1;16(2):e0244860. doi: 10.1371/journal.pone.0244860 (PMC7850494; doi:10.1371/journal.pone.0244860)
Supplement: S1 Table — (PDF) [file pone.0244860.s005.pdf]

S1 Table. Survey for the collection of basic demographic information from users of the app.

Question	Responses	
About how long have you been using the app (in months)? (Select 0 if you are a new user.)	Range: 0-100	
What is your level of medical training?	Physician: Attending/Consultant<br>Physician: Fellow/Resident/Registrar<br>Anesthesia Assistant (PA)<br>Nurse Anesthetist (CRNA)<br>Nurse (RN)<br>Technically Trained in Anesthesia<br>Anesthesia Technician<br>Student AA	Student Nurse Anesthetist<br>Medical Student<br>Paramedic/EMT<br>I am not a medical practictioner<br>Respiratory Therapist<br>Pharmacist<br>Other type of medical provider
What are your medical specialties?	Anesthesiology<br>Pain Medicine<br>Pediatric Anesthesiology<br>Cardiothoracic Anesthesiology<br>Obstetric Anesthesiology<br>Adult Critical Care	Pediatric Critical Care<br>Emergency Medicine<br>Pediatrics<br>Internal Medicine<br>Other
How many years have you been in practice? (Not counting years in training)	Range: 0-50	
Please rate the importance of the app to your practice:	Not Important At All<br>Of Little Importance<br>Of Average Importance<br>Very Important<br>Absolutely Essential	
I have used the app as a reference under emergent/urgent circumstances:	True/False<br>False	
We would appreciate knowing how the app was useful to you in an emergency:	Free Text Response	
I have used the Society for Pediatric Anesthesia Critical Events Checklist:	True/False	
We would appreciate knowing how the checklist was useful to you:	Free Text Response	
What are is your practice size?	I am the only practitioner for large area<br>One of several practitioners in the area<br>Group practice 1-5 members<br>Group 5-10 members	Group 10-25 members<br>Group 25-50 members<br>Group > 50 members
What is your anesthesia practice model?	Physician only<br>Physician supervised, anesthesiologist on site<br>Physician supervised, non-anesthesiologist physician on site	Physician supervised, no physician on site<br>No physician supervision<br>Not an anesthesia provider
What is your primary practice environment?	Private clinic or office<br>Local health clinic<br>Ambulatory surgery center	Small community hospital<br>Large community hospital<br>Academic department/University hospital
What is community does your practice primarily serve?	Rural<br>Suburban<br>Urban	
I use of the app as a reference for which classes of patients/procedures?	Adult<br>Pediatrics<br>Obstetric<br>Cardiothoracic<br>Intensive Care	Regional<br>Pain<br>Emergency Room<br>Other
