# Supplementary material for: Regional anesthesia educational material utilization varies by World Bank income category: A mobile health application data study
Source: PLoS One. 2021 Feb 1;16(2):e0244860. doi: 10.1371/journal.pone.0244860 (PMC7850494; doi:10.1371/journal.pone.0244860)
Supplement: S2 Table — (PDF) [file pone.0244860.s006.pdf]

S2 Table. Raw Data Used to Create Choropleth (Figure 1)

Percentage of users clicking nerve block	ISO-3166 alpha-2 code	All Responses	World Bank Income
9.549071618	AE	377	High income
13.29879102	AF	579	Low income
10	AG	10	High income
13.23529412	AL	136	Upper middle income
14.94252874	AM	87	Upper middle income
11.02941176	AO	272	Lower middle income
14.43633414	AR	1233	Upper middle income
20	AS	5	Upper middle income
8.525754885	AT	563	High income
9.677419355	AU	496	High income
57.14285714	AW	7	High income
12.5	AZ	208	Upper middle income
7.112970711	BA	239	Upper middle income
13.33333333	BB	15	High income
10.67044381	BD	1059	Lower middle income
10.14492754	BE	552	High income
19.62616822	BF	107	Low income
13.67673179	BG	563	Upper middle income
12.30769231	BH	65	High income
20.10050251	BI	199	Low income
12.90322581	BJ	62	Low income
18.18181818	BN	11	High income
15.39548023	BO	708	Lower middle income
15.36358666	BR	3658	Upper middle income
12.5	BS	16	High income
13.04347826	BT	46	Lower middle income
10.63829787	BW	47	Upper middle income
10.82004556	BY	878	Upper middle income
17.5	BZ	40	Upper middle income
8.438061041	CA	557	High income

10.74856046	CD	521	Low income
11.29032258	CG	62	Lower middle income
7.746478873	CH	426	High income
15.22988506	CI	348	Lower middle income
11.88369153	CL	791	High income
10.95571096	CM	429	Lower middle income
19.73273942	CN	2245	Upper middle income
9.213661636	CO	2518	Upper middle income
5.333333333	CR	75	Upper middle income
8.631921824	CU	614	Upper middle income
25	CV	8	Lower middle income
8.823529412	CY	68	High income
9.6	CZ	750	High income
9.136420526	DE	5593	High income
12.5	DJ	48	Lower middle income
13.63636364	DK	110	High income
8.823529412	DO	442	Upper middle income
12.92134831	DZ	2314	Upper middle income
12.96958855	EC	1118	Upper middle income
6.25	EE	96	High income
10.68190777	EG	2537	Lower middle income
50	ER	2	Low income
11.09271523	ES	2416	High income
13.372582	ET	1189	Low income
16.66666667	FI	78	High income
12.61261261	FJ	111	Upper middle income
10.68925234	FR	1712	High income
5.454545455	GA	55	Upper middle income
8.747855918	GB	1166	High income
21	GE	300	Upper middle income
11.37905049	GH	1327	Lower middle income
9.230769231	GM	65	Low income
9.210526316	GN	76	Low income
7.555555556	GR	450	High income
14.09395973	GT	149	Upper middle income

11.00917431	GY	109	Upper middle income
5.405405405	HK	111	High income
11.72413793	HN	145	Lower middle income
13.67837338	HR	541	High income
10.77844311	HT	167	Low income
11.87683284	HU	682	High income
8.236239454	ID	4978	Lower middle income
11.15537849	IE	251	High income
17.57188498	IL	626	High income
11.69141194	IN	17175	Lower middle income
11.50729335	IQ	1234	Upper middle income
5.594785443	IR	1841	Upper middle income
6.666666667	IS	30	High income
14.90864799	IT	4105	High income
5	JM	100	Upper middle income
14.32291667	JO	384	Upper middle income
5.882352941	JP	170	High income
19.75036711	KE	1362	Lower middle income
10.09174312	KG	109	Lower middle income
14.79289941	KH	169	Lower middle income
25	KI	4	Lower middle income
11.9047619	KM	42	Lower middle income
50	KN	2	High income
6.069364162	KR	346	High income
9.803921569	KW	255	High income
50	KY	6	High income
17.36263736	KZ	455	Upper middle income
10.20408163	LA	196	Lower middle income
12.28813559	LB	236	Upper middle income
25	LC	4	Upper middle income
10.16260163	LK	246	Upper middle income
7.947019868	LR	151	Low income
22.22222222	LS	36	Lower middle income
12.17391304	LT	230	High income
6.666666667	LU	30	High income

10.43956044	LV	182	High income
7.598039216	LY	1224	Upper middle income
13.17296678	MA	873	Lower middle income
25	MC	4	High income
16.16766467	MD	167	Lower middle income
6.060606061	ME	33	Upper middle income
6.981981982	MG	444	Low income
12.5	MK	176	Upper middle income
12.5	ML	232	Low income
5.911330049	MM	406	Lower middle income
10.90909091	MN	110	Lower middle income
12.5	MO	8	High income
11.47540984	MR	61	Lower middle income
10.52631579	MT	19	High income
10.47120419	MU	191	Upper middle income
11.42857143	MV	35	Upper middle income
11.53846154	MW	78	Low income
11.34674246	MX	4574	Upper middle income
5.591524426	MY	1699	Upper middle income
7.619047619	MZ	105	Low income
8.24742268	NA	97	Upper middle income
12.5	NC	8	High income
12.75167785	NE	149	Low income
12.6493324	NG	1423	Lower middle income
11.11111111	NI	198	Lower middle income
6.41025641	NL	858	High income
10.27667984	NO	253	High income
12.90801187	NP	674	Low income
2.380952381	NZ	84	High income
4.861111111	OM	144	High income
10.31746032	PA	126	High income
10.75201989	PE	1609	Upper middle income
21.48148148	PG	135	Lower middle income
7.861425716	PH	1501	Lower middle income
10.6639839	PK	3976	Lower middle income

9.501702259	PL	3231	High income
13.7254902	PR	102	High income
8.943089431	PS	246	Lower middle income
8.951965066	PT	1374	High income
10.15384615	PY	325	Upper middle income
11.84210526	QA	152	High income
10.87239583	RO	1536	Upper middle income
11.32478632	RS	468	Upper middle income
12.96122869	RU	5339	Upper middle income
15.63342318	RW	371	Low income
13.46282652	SA	1493	High income
9.090909091	SB	22	Lower middle income
11.11111111	SC	18	High income
7.804370447	SD	961	Lower middle income
11.23287671	SE	365	High income
4.347826087	SG	92	High income
9.677419355	SI	589	High income
9.638554217	SK	332	High income
12.32876712	SL	73	Low income
21.80451128	SN	133	Lower middle income
7.028753994	SO	313	Low income
5.555555556	SR	18	Upper middle income
9.090909091	SS	33	Low income
28	ST	25	Lower middle income
4.62633452	SV	281	Lower middle income
13.15789474	SY	532	Low income
14.28571429	SZ	21	Lower middle income
4.761904762	TD	84	Low income
23.52941176	TG	34	Low income
8.142493639	TH	393	Upper middle income
9.009009009	TJ	111	Low income
21.42857143	TL	14	Lower middle income
15.73033708	TM	89	Upper middle income
7.535321821	TN	637	Lower middle income
25	TO	8	Upper middle income

12.31588844	TR	3191	Upper middle income
6.666666667	TT	105	High income
7.142857143	TW	238	High income
14.33597186	TZ	1137	Low income
12.43243243	UA	1665	Lower middle income
21.89349112	UG	338	Low income
9.539327507	US	6751	High income
10.37037037	UY	135	High income
11.63895487	UZ	421	Lower middle income
8.5995086	VE	814	Upper middle income
10.29411765	VN	680	Lower middle income
28.57142857	VU	7	Lower middle income
9.195402299	XK	87	Upper middle income
14.47876448	YE	1036	Low income
11.79723502	ZA	1085	Upper middle income
14.85714286	ZM	175	Lower middle income
8.064516129	ZW	310	Lower middle income
