# Supplementary material for: Regional anesthesia educational material utilization varies by World Bank income category: A mobile health application data study
Source: PLoS One. 2021 Feb 1;16(2):e0244860. doi: 10.1371/journal.pone.0244860 (PMC7850494; doi:10.1371/journal.pone.0244860)
Supplement: Attachment — Submitted filename: Response to Reviewers.docx [file pone.0244860.s007.docx]

Dear Professor Mandell,

We appreciate the opportunity to submit a revised version of our manuscript entitled: “Evaluating worldwide interest in regional anesthesia educational resources: an observational study of mobile health application data” (JPONE-D-20-25898).

We have reviewed yours and the referees’ comments and responded to concerns both in this letter and via changes to the manuscript. All comments have been addressed.

This feedback helped to clarify and strengthen the manuscript, and we hope that our modifications will be satisfactory to you and to the reviewers. We would welcome any additional feedback. Please do not hesitate to contact me with questions, concerns, or other changes that might be necessary prior to final publication.

Regards,

Vanessa Moll, MD, PhD, FCCM, FASA

**Revision Notes:**

1. Editors comment 1: I am providing editorial comments about limitations of this study that should be addressed. It was not clear what specific problems the investigators are studying, who this is important to and what gap in knowledge this study fills. These points are essential building blocks that should be clearly articulated in the Introduction of every scientific paper so the readers can understand why the study was done. The contextual framing for this study is therefore underdeveloped. Readers need this information to understand how this study will advance their knowledge and improve medical care. Can the authors please revise the paper to make these points clear.

Thank you for the opportunity to clarify. We rewrote the Introduction (including abstract introduction) to reflect these suggestions.

2. Editors comment 2: There are a number of biases in this study that have not been fully explored. I share the critique brought up by the reviewers regarding the use of Android as the sole source of the study data. It is also possible that providers used other Android applications. The authors examined a study sample and not a census. All samples need to be well defined; i.e. how the sample relates the whole population. The authors would have to cite how many healthcare providers from LMIC use Android compared to other types of smart phones or even those with no access to this technology. How do these populations compare to each other? Do they have the same resources and approach to treatment? It is unlikely this data is available so the authors should acknowledge this as an important limitation to their study. It is problematic the authors are studying a section of the healthcare population that they are unable to define in comparison to the whole. Other important sources of bias the authors have not discussed thoroughly include a lack of information on how many healthcare providers with potential access did not use this resource. Once again, I anticipate this information may be almost impossible to access but the issue deserves more attention. The authors are obliged to explain that the information in this study cannot be generalized and limits the clinical utility of the data.

Thank you for these suggestions, and we agree that these are critical considerations that warrant clarification in the manuscript to better frame our work. We incorporated the following into the manuscript:

Gathering data about health or healthcare information-seeking via mobile health apps is feasible, timely, and cost-effective to deploy at scale [[40]](https://paperpile.com/c/tU6jOL/xK3Ah). Mobile networks are advantageous in areas with limited landline infrastructure. For instance, in 2015 there were 5.5 billion mobile phone subscriptions in developing countries, representing nearly 92 subscriptions per 100 inhabitants [[37]](https://paperpile.com/c/tU6jOL/azgHC). Mobile applications therefore offer an opportunity to better understand clinical practice, information-seeking behavior, and other behaviors relevant to health services research within LMIC.

Our application is exclusive to the Android platform, which likely serves to introduce selection bias. However, Android is estimated to have greater global penetration compared to other mobile operating systems, which may serve to capture both more users and a greater proportion of users outside of high-income countries [[14]](https://paperpile.com/c/tU6jOL/wZDYd). We examined user behavior of only one mobile health application, and our findings are therefore specific to this particular subset of the greater healthcare workforce. As such, the generalizability of our findings beyond this subset is limited. Among users of the application, differences in participant income, consistency or quality of internet access, parallel utilization of offline reference material, local practice patterns, or other unaccounted-for variables are all important factors that may serve to introduce confounding. Within the context of these important limitations, our findings offer novel insight into clinical practice patterns that might not otherwise be possible on a global scale via alternative methodologies.

3. Editors comment 3: Can the authors provide some type of link to the “Anesthesiologist”? The authors should include a discussion about the “evidence/validity” for crowd sourced educational materials. Is this application the Wikipedia of anesthesia practice? Is there any peer review of the materials?

We added the following to the supplement:

Description of the app Anesthesiologist

The app “Anesthesiologist” was designed to help quickly calculate adult and pediatric anesthesia related information like common drug dosing or airway related information such as endotracheal tube size. The app was written solely to be used as a helpful adjunct for professionally trained physicians and practitioners otherwise experienced in airway management and drug administration and dosing. There is no peer review of the app in the classical sense; the app functions much as a calculator and not as guidelines. The anesthesiologist app does provide crowd sourced educational material in the form of links to nerve blocks available publicly on YouTube (videos peer-reviewed by the app’s author) and it crowdsources information by asking professionals to voluntarily fill out surveys.

The app can be found here: <https://play.google.com/store/apps/details?id=com.shahlab.anesthesiologist&hl=en_US>

We also added a section discussing crowd-sourced education or free online medical educational resources in the discussion:

The use of social media in regional anesthesia education

This study is utilizing crowd-sourced educational videos describing different ultrasound guided nerve blocks. Especially, in recent years the use of social media and the advent of free online medical education (FOAM) has grown substantially in medicine and specifically in regional anesthesia with its benefits and challenges described in detail elsewhere [[28,29]](https://paperpile.com/c/tU6jOL/QIkW+yMKW). Although these crowd-sourced resources need to be met with a healthy amount of scepticism, the sharing of videos and images especially in ultrasound guided procedures has led to improved education in regional anesthesia [[28]](https://paperpile.com/c/tU6jOL/QIkW). In anesthesiology, the use of FOAM and digital platforms has allowed for greater accessibility , portability and flexibility in medical education [[30]](https://paperpile.com/c/tU6jOL/1fTt). A recent review even argued that the “future of medical education is social (media)” [[31]](https://paperpile.com/c/tU6jOL/np6W)Crowd-sourced educational materials are not a substitute for textbooks and clinical training, but are becoming a bigger part of the education for both novice and experienced regional anesthesiologists with benefits such as global interaction and knowledge translation within the specialty. With the growing FOAM movement there is now an unprecedented ability to share information beyond traditional venues (e.g. conferences or publications) which might have an impact especially in low-resourced countries [[32–35]](https://paperpile.com/c/tU6jOL/tLV6+gx2h+7gBi+aNVZ).

4. Editors comment 4: The discussion contains a considerable amount of speculation and theorizing based on little data. I ask the authors to revise this section to explore the strengths and weaknesses of their data. What do their findings mean? It is not helpful to speculate that certain blocks may be used because of more traffic accidents when there is no data in this or other studies to support this statement. But the authors can present this as a question that needs further evaluation in another study.

Thank you for these suggestions. We eliminated our reference to road traffic accidents and realigned the discussion.

5. Reviewer 1, comment 1: A distribution of the countries/ regions from which responses were elicited would have indicated that this was truly a study which reflected worldwide interest. Since an income based analysis has been carried out the title does not adequately reflect that this was primarily an analysis based on income levels and app utilization.

We changed the title to the following: Evaluating global interest in regional anesthesia educational resources based on income levels: an observational study of mobile health application data

6. Reviewer 1, comment 2: Practitioners in LMIC accessed the app more frequently (22,062 clicks) than in HIC (8,409 140 clicks). – It appears that this refers to the usage of the App. Since the App provides other information eg. Drug dose calculation, it may be pertinent to compare access of Nerve block related material eg. tutorials specifically.

Thank you for this comment we clarified in the Results: Practitioners in LMIC accessed the linked nerve block videos more frequently (22,062 clicks) than in HIC (8,409 clicks).

7. Reviewer 1, comment 3: “LMIC users had the highest in-app clicks for surgical peripheral nerve blocks” Since the results may be used for designing educational tools based on the unique requirements of each group, it might be useful to consider size of practitioner population in each of these categories (LMIC, HIC) and give appropriate weightage when considering the utilization of the App and its links as well as in arriving at conclusions.

We have corrected the statement to read "LMIC users had the highest *rate* of in-app clicks for surgical peripheral nerve blocks" We agree that the specific subpopulations of providers will differ between LMIC and HIC, but it is not clear how to modify or adjust our presented findings on the basis of the size of the practitioner population and the distribution of the subtypes of providers (e.g. attendings, trainees, non-physicians). This difficulty led to our decision to provide unadjusted usage rates, which was the most straightforward way to interpret the incoming data. In addition, while we agree that educational tools should be tailored to individual needs, the design of these tools is beyond the scope of this article. However, this feature could be incorporated into future versions of this app.

8. Reviewer 1, comment 4 :“Postoperative pain blocks were clicked at a higher percentage (2715/32.3%) in HIC compared to low (521/21.6%), lower middle (2090/21.4%), and 145 upper middle-income countries (2112/21.4%)”.

Maybe pertinent to consider the effect of confounding factors such as local policy relating to post operative pain relief that may have produced the above result.

The level of significance of the difference in App utilization between different income regions is not clearly explained.

Thank you for this comment. Given the limitations of this dataset (i.e., single app, Android platform), we did not perform any statistical analysis and present the data as purely descriptive. We agree with the reviewer that many factors may affect clinical practice at the institutional level and have added local policy as a confounding factor to the limitations of the study: Therefore, differences in participant income, lack of access to the internet or other unaccounted-for variables such as local policy or standards for analgesic regimens could skew the app user base and introduce confounding.

9. Reviewer 2, comment 1: the data collection done can be interpreted as being one sided. The authors discuss how their collection of data may have answered their question, but they do fail to report on issues that may make the data collected less significant. Although they discuss the use of an app (that apparently just has links to youtube videos) they fail to simply comment and discuss the fact that it is an android app. One can assume that there is going to be a variance in the HIC to the LIC in terms of the use of android devices to non android devices. Does the app only work on android devices or can it be used on non-android devices as well? If that is the case, the introduction can lead to a little confusion. If it is that the app cannot, then the data might be slightly different if that unmentioned data is included. Meaning, those without androids how to learn and what are they searching for if looking up these videos on youtube directly? However that is not discussed and it is not entirely clear, in my eyes. In many HIC, like the U.S. and Eastern Europe, non-android devices may be utilized more frequently. Also, five years of data collection with today's technology may have data that may favor prior years to later years and vice-versa.

Thank you for these comments. The app is solely offered for Android platforms, but Android applications are estimated to have 74% global market share penetration with higher use in LMICs. We now discuss the limitations to an Android only app. Please refer to our answer to Editors comment 3 for details.

10. Reviewer 2, comment 2: It is nice that the effect of COVID-19 is discussed, but is the data discovered simply because case loads were down or because people stopped looking or both or other? Would be interesting to comment about in the discussion.

In a separate study we specifically sought to determine whether the anaesthesiology app data could serve as a qualitative proxy for global surgical case volumes and therefore had the ability to monitor the impact of the coronavirus disease 2019 pandemic.

We added the following sentence to provide an explanation to the reviewers comment:

In a separate study, App data provided a proxy for surgical case volumes, and could therefore be used as a real-time monitor of the impact of COVID-19 on surgical capacity [[13]](https://paperpile.com/c/qAirw3/OyGo).

11.	Reviewer #3: well written ,insightful , the material is very relevant to current standards of practice in both academic and private group setting. I am recommending this material for publishing with no reservations

Thank you.

**Journal Requirements:**

When submitting your revision, we need you to address these additional requirements.

1. Please ensure that your manuscript meets PLOS ONE's style requirements, including those for file naming. The PLOS ONE style templates can be found at

https://journals.plos.org/plosone/s/file?id=wjVg/PLOSOne_formatting_sample_main_body.pdf and

<https://journals.plos.org/plosone/s/file?id=ba62/PLOSOne_formatting_sample_title_authors_affiliations.pdf>

We ensured proper style and naming requirements.

2. We note that Figure 1 in your submission contain map images which may be copyrighted. All PLOS content is published under the Creative Commons Attribution License (CC BY 4.0), which means that the manuscript, images, and Supporting Information files will be freely available online, and any third party is permitted to access, download, copy, distribute, and use these materials in any way, even commercially, with proper attribution. For these reasons, we cannot publish previously copyrighted maps or satellite images created using proprietary data, such as Google software (Google Maps, Street View, and Earth). For more information, see our copyright guidelines: http://journals.plos.org/plosone/s/licenses-and-copyright.

We require you to either (1) present written permission from the copyright holder to publish these figures specifically under the CC BY 4.0 license, or (2) remove the figures from your submission:

This figure was created by one of the authors using the tmap package for R as described in Methods.

3. Please include captions for your Supporting Information files at the end of your manuscript, and update any in-text citations to match accordingly. Please see our Supporting Information guidelines for more information: http://journals.plos.org/plosone/s/supporting-information.

We have updated the captions for Supporting Information at the end of the manuscript.
